# Diagnostic Potential of CD44, CD133, and VDR in Epithelial Ovarian Tumors: Association with Histopathology Parameters

**DOI:** 10.3390/ijms26083729

**Published:** 2025-04-15

**Authors:** Ljubiša Jovanović, Branka Šošić-Jurjević, Anđa Ćirković, Sandra Dragičević, Branko Filipović, Svetlana Milenković, Stefan Dugalić, Miroslava Gojnić-Dugalić, Aleksandra Nikolić

**Affiliations:** 1 Department of Pathology and Medical Cytology, University Clinical Center of Serbia, dr Koste Todorovića 26, 11000 Belgrade, Serbia; 2 Department of Cytology, Institute for Biological Research “Siniša Stanković”, National Institute of Republic of Serbia, University of Belgrade, Bulevar Despota Stefana 142, 11108 Belgrade, Serbia; 3 Institute for Medical Statistics and Informatics, Faculty of Medicine, University of Belgrade, dr Subotića 15, 11000 Belgrade, Serbia; 4 Laboratory for Molecular Biology, Institute of Molecular Genetics and Genetic Engineering, University of Belgrade, Vojvode Stepe 444a, 11000 Belgrade, Serbia; 5 Clinic for Gynecology and Obstetrics, University Clinical Center of Serbia, dr Koste Todorovića 26, 11000 Belgrade, Serbia; 6 Faculty of Medicine, University of Belgrade, dr Subotića 8, 11000 Belgrade, Serbia

**Keywords:** cancer stem cells, ovarian cancer, immunohistochemistry, CD44, CD133, vitamin D receptor

## Abstract

Cancer stem cells (CSCs) significantly contribute to heterogeneity, malignancy, and therapy resistance in ovarian cancer. Recent studies emphasize the role of the vitamin D receptor (VDR) in regulating cell differentiation and stemness in various types of cancer. This study aims to determine the expression levels of CD44, CD133, and VDR in epithelial ovarian tumors (EOTs) and to compare these levels across different tumor types, including benign, atypical proliferative tumors, and five types of malignant phenotypes, in order to evaluate their potential as diagnostic tools for malignancy. Tissue samples from 218 patients diagnosed with EOT were analyzed. Clinical and histopathologic parameters were recorded. Quantitative immunohistochemical tissue microarray analysis was used to assess the expression levels of CD44, CD133, and VDR using two different scoring systems. Comparisons were made between benign tumors (n = 45), atypical proliferative tumors (n = 42), and ovarian carcinomas (n = 131), including high-grade serous (HGSC) and non-HGSC subtypes. Ovarian cancer, especially HGSC, showed a significantly higher expression of CD44 and VDR (*p* < 0.05) compared to atypical proliferative tumors and benign tumors. The expression of CD133 was highest in atypical proliferative tumors (*p* < 0.05). A moderate positive correlation was found between CD44, CD133, and VDR in all groups, with significant correlations with tumor grade and FIGO stage in ovarian cancer (*p* < 0.05). The increased expression of CD44 and VDR in aggressive ovarian cancer, along with elevated CD133 levels in atypical proliferative tumors, highlights the complexity of tumor biology. These markers may serve as valuable targets for the diagnosis of ovarian cancer.

## 1. Introduction

Epithelial ovarian tumors (EOTs) represent a significant health concern, often associated with poor prognosis due to late-stage diagnosis and treatment resistance. EOTs are characterized by considerable heterogeneity, which complicates treatment strategies. Ovarian cancers (OCs) are classified into five primary phenotypes: high-grade serous cancer (HGSC), low-grade serous cancer, endometrioid, clear cell, and mucinous cancer [1]. HGSC is the most aggressive and lethal malignancy in gynecological cancers, often becoming resistant to standard chemotherapy treatments, with an overall 5-year survival rate of approximately 30–40% [2]. Approximately 250,000 women per year worldwide are diagnosed with OC [3].

The clinical behavior of ovarian cancers exhibits significant variability based on the cell of origin, suggesting a critical role for cancer stem cells (CSCs) in progression, growth, metastasis, and resistance to chemotherapy [4,5]. It is generally assumed that CSCs can be recognized by various surface markers and/or combinations of markers, including CD44+, CD24+, CD117+, CD133+, and ALDH1+ [6,7,8]. CD44 is a transmembrane glycoprotein with a significant role in intercellular interactions involving cell migration and proliferation [9,10,11]. CD133 (prominin-1) is a membrane glycoprotein expressed in CSCs of many cancer types [12,13,14], and it is localized in membrane protrusions of the apical polarity of epithelial cells [15,16]. Considering the widespread functions of CSCs regulated by CD133, it is a useful marker for their detection [16]. Some studies have highlighted the importance of high immunohistochemical expression levels of CD133 (prominin-1) as a marker of aggressive tumor characteristics and poorer clinical outcomes [12,13,16,17], while others have identified CD44-positive cells as critical players in tumor progression, influencing both tumor behavior and patient prognosis [18,19,20]. Researchers are currently examining the correlations between clinical outcomes in EOT patients and the phenotypic characteristics of CSCs [21].

The vitamin D receptor (VDR) is a nuclear receptor that mediates the effects of 1,25-dihydroxyvitamin D3. It is ubiquitously expressed in human tissues, and vitamin D-VDR-mediated signaling is crucial for the regulation of calcium homeostasis, as well as immunity, cell growth and differentiation, and energy metabolism [22,23]. Epidemiologic evidence indicates that adequate vitamin D levels are associated with a lower risk of OCs and reduced cancer mortality [24]. In line with this, several reports suggest vitamin D as a potential therapeutic agent for OC treatment [25]. More recent studies have shown increased immunohistochemical VDR expression in gynecological and ovarian cancers [26,27,28]. This is linked to changes in cancer cell differentiation and stemness, influenced by interactions of VDR-mediated signaling with other growth-promoting factors or microRNAs [27,29,30,31].

To our knowledge, no comparative or cross-sectional studies have examined the expression of potential CSC markers alongside VDR in a cohort of patients with epithelial ovarian tumors (EOTs) exhibiting varying pathological features. The combination of CD44, CD133, and VDR as potential diagnostic markers in ovarian cancer is supported by their roles in cancer stem cell biology, their association with aggressive tumor characteristics, and their influence on clinical outcomes. This study aims to analyze differences in immunostaining intensity for CD44, CD133, and VDR among groups of benign tumors, low-grade atypical proliferative tumors, and five distinct malignant phenotypes (HGSC and non-HGSC). Additionally, we assess the association between these expression markers, tumor grade, and FIGO stage. This combination is expected to enhance diagnostic features, facilitate the identification and characterization of targeted therapies, and ultimately lead to improved patient outcomes.

## 2. Results

### 2.1. Patients

Patients’ characteristics and tumor phenotypes are presented in Table 1. Based on the analysis of 218 ovarian tumor patients, ovarian cancer was diagnosed in 131 patients (60%), atypical proliferative tumors in 42 patients (19%), and benign ovarian tumors in 45 patients (21%). There was a significant increase (*p* < 0.001) in the prevalence of ovarian cancer in older and postmenopausal women compared to those with atypical proliferative tumors (APTs) or benign ovarian tumors (BOTs) (Table 1). The majority of cancers had bilateral localization (84%, *p* < 0.001), while this localization was rare in APT (14%) and benign ovarian tumors (11%). The average maximum tumor diameter differed significantly between the study groups: it was largest in the APT group (*p* < 0.001) and smallest in the benign ovarian tumor group (*p* < 0.001). There was no significant difference in the maximum tumor diameter between ovarian cancers and benign ovarian tumors (Table 1).

Among the patients with ovarian cancer (OC), we observed five histological types (Table 2): the most common was high-grade serous cancer (HGSC; 63.4%), followed by low-grade serous cancer (LGSC; 16.8%), mucinous (5.3%), endometrioid (6.1%), and clear cell cancer (8.4%) (Table 2). The most common non-HGSC ovarian cancer was LGSC, while the rarest was mucinous ovarian cancer. Nearly half of all cancers were diagnosed in FIGO stages I and III (45% and 47.3%), while stages II and IV were much less common (6.9% and 0.8%, respectively). Most ovarian cancers had a differentiation grade of III (60.3%). More than half of the ovarian cancers exhibited lymphovascular invasion, necrosis, intratumoral, and peritumoral lymphocytic infiltration. Specifically, OC cases were characterized by the presence of lymphovascular invasion (64.1%), necrosis (61.1%), intratumoral lymphocytic infiltration (64.9%), and peritumoral lymphocytic infiltration (73.3%) (Table 2).

### 2.2. CD44, CD133, and VDR Expressions in Epithelial Ovarian Tumors

**Figure 1:**
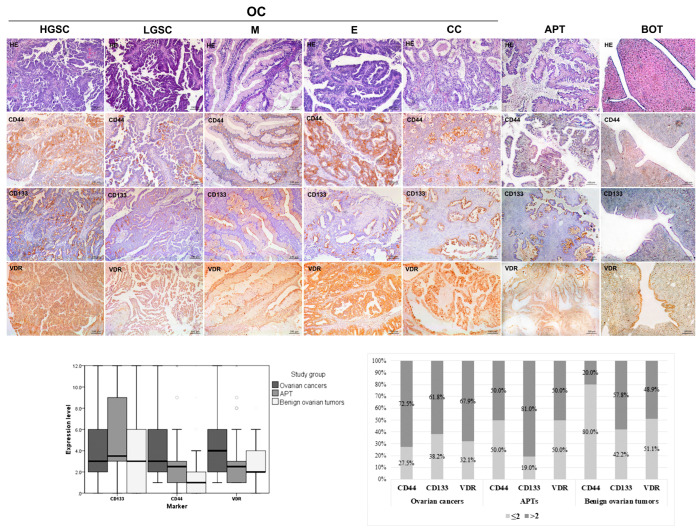
Representative micrographs (×100) of immunohistochemical staining of CD44, CD133, and VDR in ovarian cancers (high-grade serous cancer, HGSC; low-grade serous cancer, LGSC; mucinous, M; endometrioid, E; and clear cell, CC), atypical proliferative tumors (APTs), and benign ovarian tumors (BOTs); scale bar, 100 µm. Quantification analysis for expression levels of CD44, CD133, and VDR was performed using the IR score, and the results are presented as total expression levels (mean ± standard deviation; left chart), and percentages (%) of high (>2) and low (<2) expression levels for each tumor phenotype (n = 218; right chart).

The immunohistochemical analysis of all histological types of EOT demonstrated varying levels of expression for the examined CSC markers CD44, CD133, and VDR (Figure 1). Statistical analysis revealed that the overall CD44 expression level was significantly higher in OCs compared to APTs (*p* = 0.001) and BOTs (*p* < 0.001). Additionally, APTs exhibited a higher CD44 expression level than BOTs (*p* < 0.001; Figure 1). In contrast to CD44, the expression of CD133 was not significantly increased in high-grade ovarian cancers compared to APTs (*p* = 0.157) or BOTs (*p* = 0.060). The overall VDR expression level was significantly higher in OCs than in APTs (*p* = 0.001) and BOTs (*p* = 0.003), while there was no significant difference between APTs and BOTs (*p* = 0.610) (Figure 1).

Next, we analyzed the high expression level (>2) of examined CSC markers and VDR in all examined types of EOTs (Figure 1). In line with the total expression levels of the examined markers, OCs were characterized by significantly higher levels of CD44-immunostaining intensity compared to APTs and BOTs (*p* = 0.007 and *p* < 0.001, respectively), and APTs had higher levels than BOTs did (*p* = 0.003). A high expression of CD133 was more common in APTs than in OCs and BOTs (*p* = 0.022 and *p* = 0.020, respectively), while there was no difference in frequency of the high expression of CD133 between OCs and BOTs (*p* = 0.631). High VDR expression (>2) was more common in OCs than in APTs and BOTs (*p* = 0.036 and *p* = 0.022, respectively), while there was no significant difference in the frequency of high VDR expression between APTs and BOTs (*p* = 0.918).

### 2.3. CD44, CD133, and VDR Expression Levels in Ovarian Cancers

**Figure 2:**
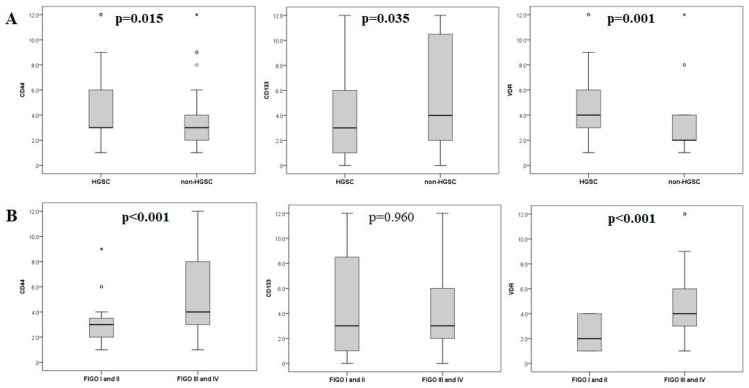
The expression levels of CD44, CD133, and VDR (**A**) according to ovarian cancer histological type (HGSC vs. non-HGSC) and (**B**) FIGO stage (Figo I and I vs. FIGO III and IV); circles and stars represent outliers and extreme values, respectively (n = 131).

Expression levels of CD44, CD133, and VDR according to histological type (HGSC vs. non-HGSC) and FIGO stage (FIGO I and II vs. FIGO III and IV) are presented in Figure 2. There were significantly higher levels of CD44, CD133, and VDR expression in HGSC than in non-HGSC ovarian cancers (*p* = 0.015, *p* = 0.035, and *p* = 0.001, respectively) (Figure 2A). The expression level of CD44 and VDR was significantly higher in ovarian cancers with FIGO stages III and IV than in FIGO stages I and II (*p* < 0.001 and *p* < 0.001, respectively), while the expression level of CD133 showed no difference between lower (I and II) and higher (III and IV) FIGO stages (*p* = 0.267) (Figure 2B).

**Figure 3:**
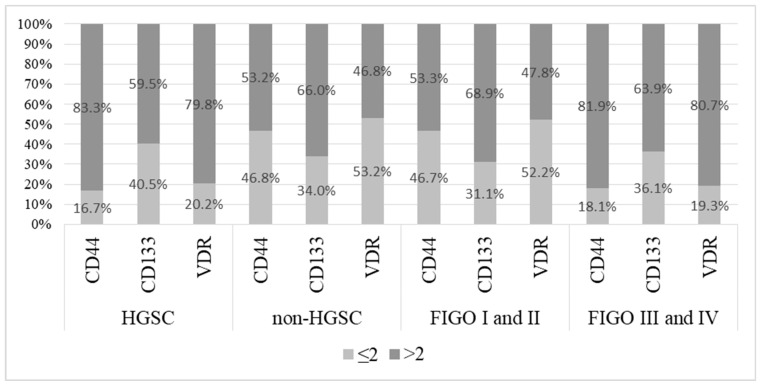
The distribution of the high-expression category (>2) of CD44, CD133, and VDR in ovarian cancers (n = 131) according to histological type and FIGO stage. Quantification analysis for expression levels of CD44, CD 133, and VDR was performed using the IR score. The results are presented as percentages (%) of high (>2) and low (<2) expression levels using the IR score for each tumor phenotype.

The distribution of the high-expression category (>2) for CD44, CD133, and VDR according to histological type (HGSC vs. non-HGSC) and FIGO stage (FIGO stages I and II vs. FIGO stages III and IV) is presented in Figure 3. There were significantly more cases with a high expression level (>2) of CD44 and VDR in HGSC than in non-HGSC ovarian cancers (*p* < 0.001 and *p* < 0.001, respectively), while there was no difference in the distribution of the high-expression category of CD133 between HGSC and non-HGSC ovarian cancers (*p* = 0.467). A similar result was obtained when comparing ovarian cancers with lower (I and II) and higher (III and IV) FIGO stages. There were significantly more cases with high expression levels (>2) of CD44 and VDR in ovarian cancers with higher FIGO stages (III and IV) than in those with lower FIGO stages (I and II) (*p* < 0.001 and *p* < 0.001, respectively), while there was no difference in the distribution of the high-expression category of CD133 between ovarian cancers with lower and higher FIGO stages (*p* = 0.484).

Finally, we examined the nonparametric Spearman’s correlation between the expressions of the examined CSC markers, VDR, cancer grade, and FIGO stage in OCs. All results are summarized in Table 3. We determined the moderately positive statistically significant association between the cancer grade and CD44 (r = 0.3930, *p* < 0.0001), VDR (r = 0.4170, *p* < 0.0001), and FIGO stage (0.3875, *p* < 0.0001). Regarding the correlation between the examined expression markers and FIGO stage, there was a moderately positive statistically significant association between CD44 and FIGO (r = 0.4028, *p* = 0.0001) and VDR and FIGO (r = 0.6496, *p* < 0.0001), whilst there was no association between CD133 and cancer grade (r = −0.08114, 0.3569) or FIGO stage (r = −0.07031, 0.4249). Correlation analysis between expression markers revealed a weakly positive statistically significant correlation between CD44 and CD133 (r = 0.2978, *p* < 0.0001), as well as a moderately positive correlation between CD44 and VDR (r = 0.6496, *p* < 0.0001) and CD133 and VDR (r = 0.4127, *p* < 0.0001).

## 3. Discussion

To our knowledge, this comparative cross-sectional study is the first to compare the expression levels of CD44, CD133, and VDR between patients with different types of epithelial ovarian tumors, including BOT, APT, and five types of OC. The results indicate that the immunostaining intensity for CD44 and VDR was highest in the most malignant OC, while the highest expression levels of CD133 were observed in low-grade atypical proliferative tumors.

Ovarian cancer was diagnosed in 63% of our patients, with a significant increase in occurrence among older postmenopausal women compared to those with APT or BOT. In line with this, other researchers have shown that ovarian tumors are very rare among premenopausal women, and that the incidence of OC increases after menopause [32]. Among our patients, most cancers were bilateral, while expectedly, APT and BOT showed lower rates of bilaterality. Indeed, the most prevalent and malignant phenotype of OC, HGSC, is characterized by a high degree of invasiveness at diagnosis, often involving both ovaries [33]. This type of cancer is primarily associated with genetic mutations in TP53, BRCA1, or BRCA2 [34]. In addition to the bilaterality, we examined the average maximum tumor diameter as an important but still controversial morphological parameter. The relationship between tumor size and malignancy remains debated, with some studies suggesting that larger tumors might not always indicate higher malignancy risk [35,36], which aligns with the results obtained in this study.

For the quantification of immunostaining expression of selected markers, we applied two different scoring systems. The basic scoring system was primarily based on whole tumor tissue, featuring fewer categories and a more simplified staining calculation. In comparison, the IR score (Remmele’s score) demonstrated greater sensitivity and reliability, as confirmed by statistical analysis [27].

Numerous studies have emphasized the functions of CSC that are responsible for therapy resistance in OC [27,37,38,39,40]. Many different markers have been analyzed for CSC identification in OC, most of which are surface-expressed [18,19,20]. Their characteristic expression is primarily associated with cancer niches where CSC detection is expected, thereby reflecting the biological, clinical, and histological characteristics of each type of ovarian tumor.

Our study included EOT with different biological behaviors, and therefore we selected CD44 and CD133 as the most widely used and confirmed to be specific to distinguish CSC from other cancer cells in various tumors [10,37,41]. In addition to CD44 and CD133, we analyzed the changes in immunostaining expression of VDR and found the highest expression of CD44 and VDR in OC, particularly in HGSC. The strongest expression of CD133, however, was observed in APT. Moreover, CD44 and VDR expressions were most prominent in OC in advanced FIGO stages, a finding that was also confirmed by correlation analysis. Our results, consistent with those of other studies [42], confirm that CD44 overexpression is associated with higher tumor stages and more aggressive histological types of ovarian cancer (OC). Several studies have reported a positive correlation between significant CD44 expression and poor prognosis in OC patients [18,41]. Based on our findings, as well as existing research, CD44 may serve as a valuable diagnostic marker and a promising prognostic indicator for high-grade malignant phenotypes in OC patients.

In addition to CD44, VDR was overexpressed in OC, particularly in HGSC among all examined EOTs, in line with findings from other authors [27,37]. However, this result may be considered somewhat unexpected, as the anti-tumor activity of calcitriol and its effects on CSCs have been reported in various neoplasms [43,44]. In vitro studies showed that calcitriol can inhibit OC cell proliferation in cultured cell lines, but the precise mechanism(s) of its regulation are still a matter of debate [9,37]. The bioactive form of vitamin D, 1α,25-dihydroxyvitamin D3, exerts its effects by binding to the VDR. The localization of VDR is multifaceted, with evidence indicating its presence in the cytoplasm, nucleus, and even mitochondria [45]. This diverse localization enables VDR to interact with various signaling pathways, including Wnt, Notch, Hedgehog, and TGF-β [38,42], and to influence the localization and function of β-catenin, a key player in stem cell renewal. In this context, it is reasonable to assume that increased cytoplasmic VDR expression in the high-malignant phenotype of OC reflects genetic [29,30,46] and/or alterations of regulatory pathways within the tumor microenvironment. This increase may facilitate interactions with proteins involved in stemness, thereby influencing cancer cell behavior and treatment response. Regardless of the potential mechanisms, which extend beyond the scope of this study, our results suggest that VDR (together with CD44) may serve as a valuable diagnostic marker for the HGSC type of ovarian cancer.

CD133 marker expression was most prominent in the low-malignant APT phenotype, and its expression did not correlate with histopathology parameters. CD133 is one of the most commonly used markers for CSC detection in various tumors [41], exclusively staining the apical cell membrane [11,14]. Despite the controversial relationship between CD133 expression and patient outcomes, most studies report a positive correlation between high CD133 expression and poor prognosis [47,48]. However, some authors did not find an association between CD133 expression in HGSC and survival [38]. The inconsistency in CD133 expression patterns across studies may be attributed to the heterogeneity of CSC subpopulations, differences in methodologies, and the presence of distinct clone types. CD133 is involved in various regulation pathways and its expression is influenced by different molecules, which complicates the interpretation of expression analysis results [14]. Therefore, further data are needed before it can be reliably used for diagnostic and prognostic applications [18].

While this study benefits from a large cohort size, its findings underscore the need for clinical follow-up studies to more robustly validate the prognostic relevance of the identified markers. Furthermore, targeting ovarian CSC represents an innovative therapeutic strategy. Notably, the anti-CD44 monoclonal antibody bivatuzumab, which has been investigated in head and neck squamous cell carcinoma and prostate cancer, shows promising potential for broader oncological applications [18,38,49,50]. Our findings may provide a foundation for future research aimed at exploring the mechanistic aspects of CSC/VDR interactions.

## 4. Materials and Methods

### 4.1. Patient Cohort

Our cohort comprised 218 patients with EOT including 131 with ovarian cancer (OC), 42 with atypical proliferative tumors (APTs), and 45 with benign ovarian tumors (BOTs). All patients underwent surgical resection because of their primary tumors during the three-year period. Ovarian tissue samples were obtained as part of the standard procedure for the clinical management of patients with OC. Tumor staging was performed according to the current International Federation of Gynecology and Obstetrics (FIGO) classification. Other parameters considered included patient age, menopausal status, histological type of tumor, tumor differentiation, presence of lymphovascular tumor invasion, necrosis, and lymphocyte infiltration. Patients with secondary ovarian tumors, ovarian tumors of non-epithelial origin, or those under 18 years of age were excluded from this study.

### 4.2. Histopathological Evaluation

The resected specimens were fixed in 10% buffered formalin and embedded in paraffin. For each case, the most representative block was selected. Histopathological parameters were evaluated on 4 µm thick sections stained with hematoxylin and eosin (H&E), using a Leica 2000 microscope, according to the current World Health Organization (WHO) classification (2020).

### 4.3. Tissue Microarray Construction

The ovaries were fixed in neutral buffered formalin (10%, pH 7) for 24 h. After fixation, the tissues were dehydrated in an ascending series of ethanol (30–100%) and embedded in Histowax^®^ (Histolab Product AB, Gothenburg, Sweden). The tissue microarray (TMA) was constructed from formalin-fixed and paraffin-embedded tumor samples using a 3 mm puncture needle. One core was taken from each tumor sample. The recipient paraffin block was constructed as a set of 28 cylinders [51]. Serial 5 μm thick tissue sections, obtained using a rotational microtome (RM 2125RT Leica Microsystems, Wetzlar, Germany), were deparaffinized in xylol and rehydrated in a series of decreasing ethanol concentrations (100–70%). For the histopathological evaluations, the microarray sections were stained with hematoxylin and eosin (H&E). Placental tissue, covering trophoblast and syncytiotrophoblast, served as a positive control for immunostaining [47]. Placental tissue was placed in the first row of each block for orientation.

### 4.4. Immunohistochemistry Analysis and Evaluation of Immunostaining Intensity Scoring Methods

Immunohistochemical (IHC) staining of TMA sections included the following steps: (i) After tissue deparaffinization and rehydration (2 × 5 min in xylene, followed by immersion in a series of decreasing ethanol concentrations from 100% to 70%, each for 5 min), endogenous peroxidase activity was blocked by incubating the sections with 0.3% hydrogen peroxide in methanol for 15 min. Sections were then subjected to (ii) antigen retrieval in 100 mM sodium citrate buffer (pH 6.0) and heated at 750 W in a microwave oven for 3 × 7 min. To reduce nonspecific background staining, (iii) the sections were incubated with normal goat serum (dilution 1:10 in PBS) for 45 min. Mouse monoclonal antibody (mAb) directed against human CD44 (dilution 1:100 in PBS; 156-3C11, Cell Signaling Technology, Danvers, MA, USA), rabbit mAb directed against human CD133 (dilution 1:50 in PBS; D2VBQ, Cell Signaling Technology, Danvers, MA, USA), and rabbit polyclonal VDR antibody (dilution 1:1000 in PBS; ab3508, Cambridge, MA, USA) were applied overnight at 4 °C. For the immunodetection of CD133 and VDR, the VECTASTAIN^®^ ABC (Rabbit IgG; Vector Laboratories, Newark, CA, USA) kit with the biotin/avidin system was used according to the manufacturer’s instructions. For immunodetection of CD44, goat polyclonal secondary antibody to mouse IgG-HRP (dilution 1:100 in PBS for 60 min; ab6820, Abcam, Cambridge, UK) was applied. All washes and dilutions were performed using 0.1 M PBS, pH 7.2. Binding sites were visualized using 0.05% diaminobenzidine (DAB; Serva, Heidelberg, Germany), followed by counterstaining with hematoxylin and mounting in DPX (Sigma-Aldrich, Co., St. Louis, MI, USA). Negative controls were obtained by replacing the primary antibody with PBS. Placental tissue was used as a positive tissue control and was placed in the first row of each block for orientation.

The assessment of all expressions was based on the percentage of positive tumor cells. The evaluation was performed at ×400 high microscopic magnification. Two different scoring methods were used to ensure better prognostic validity. The basic scoring system consisted of the following expression categories: negative (0), defined as no positive cells or only a single positive cell (<1%); low (1+), with less than 10% positive cells; moderate (2+), with 10–50% positive cells; and strong (3+), with more than 50% positive cells. High expression (≥10%) was defined as the combination of moderate and strong expression categories [11]. The extensive scoring method, based on specific scoring systems (IR score, Remmele’s score) [27], considered both staining intensity and the percentage of positive cancer cells. The staining intensity was scored as follows: 0 = absent, 1 = low, 2 = moderate, and 3 = strong. The percentage of positive cancer cells was scored as 0 = 0%, 1 ≤ 10%, 2 = 11–50%, 3 = 51–80%, and 4 ≥ 81%. High expression was defined as an IR score >2, while low expression was categorized as an IR score of 0–2 [27,42].

Statistical analysis using the Mann–Whitney U test and Spearman’s correlation coefficient (r) confirmed that the IR scoring method of expression analysis had better reliability. Specifically, for CD44, *p* < 0.001 and r = 0.792; for CD133, *p* < 0.001 and r = 0.836. Therefore, all results presented in this paper refer to the IR score. Immunostaining analysis of VDR was performed using the IR scoring method exclusively.

### 4.5. Statistical Analysis

Statistical analyses were performed using the Statistical Package for Social Sciences 21.0 (SPSS Inc., Chicago, IL, USA) and GraphPad Prism for Windows (v.8, San Diego, CA, USA). Numerical variables are expressed as the mean ± standard deviation (SD) or median (interquartile range, IQR), depending on the data distribution, while percentages are used for categorical variables. All data were tested for normality of distribution using the Anderson–Darling and D’Agostino and Pearson tests. Demographic and clinical parameters between study groups were analyzed using one-way ANOVA followed by Tukey’s post hoc test, the Chi-square test, or the Kruskal–Wallis test with Mann–Whitney U test, as appropriate. The association between CD44, CD133, and VDR marker expression levels and FIGO stage (I, II, III, IV) was assessed using the nonparametric Spearman’s rank correlation coefficient. The association of evaluated markers with other histopathological parameters was determined using the Chi-square test. A *p*-value of ≤0.05 was considered significant.

## 5. Conclusions

In conclusion, this comparative cross-sectional study demonstrates that the highest expression levels of the cancer stem cell marker CD44 and cytoplasmic VDR are significantly associated with aggressive histological types of EOT, particularly in patients with HGSC in FIGO stages III or IV. Our findings indicate that CD133 is not a reliable marker of tumor aggressiveness, as its expression was most prominent in APT. Future longitudinal studies are required to further assess the potential benefits of these diagnostic strategies.

## Figures and Tables

**Table 1:** Patients’ characteristics and tumor phenotypes.

Characteristics	OC n = 131	APT n = 42	BOT n = 45	OC vs. APT	OC vs. BOT	APT vs. BOT
Woman						
Age (years), Mean ± SD	60.79 ± 10.14	45.76 ± 17.25	48.69 ± 15.52	<0.001	<0.001	0.545
Menopause, n (%)	110 (84.0)	20 (47.6)	23 (51.1)	<0.001	<0.001	0.745
Tumor						
Laterality						
Unilateral	49 (37.4)	36 (85.7)	40 (88.9)	<0.001	<0.001	0.656
Bilateral	82 (62.6)	6 (14.3)	5 (11.1)			
Median diameter (mm), (min–max)	80 (18–245)	100 (30–310)	65 (3–190)	0.015	0.125	0.003

Statistical comparisons were performed using one-way ANOVA followed by Tukey’s post hoc test, as well as Chi-square tests and Kruskal–Wallis tests with Mann–Whitney U tests where appropriate, with significant differences between groups set at *p* < 0.05. Abbreviations: ovarian cancer, OC; atypical proliferative tumor, APT; benign ovarian tumor, BOT.

**Table 2:** Ovarian cancer phenotypes.

Characteristics	Description n = 131 (%)
Histological type	
HGSC	83 (63.4)
LGSC	22 (16.8)
Mucinous	7 (5.3)
Endometrioid	8 (6.1)
Clear cell	11 (8.4)
FIGO stage	
I	59 (45.0)
II	9 (6.9)
III	62 (47.3)
IV	1 (0.8)
Differentiation (Grade)	
I	43 (32.8)
II	9 (6.9)
III	79 (60.3)
Other parameters	
Lymphovascular invasion (yes)	84 (64.1)
Necrosis (yes)	80 (61.1)
Intratumor lymphocytic infiltration (yes)	85 (64.9)
Peritumor lymphocytic infiltration (yes)	96 (73.3)

Abbreviations: high-grade serous cancer, HGSC; low-grade serous cancer, LGSC.

**Table 3:** Spearman’s correlation between cancer stem cell markers CD44 and CD 133, vitamin D receptor (VDR), cancer grade, and FIGO stage in ovarian cancers.

Variables	CD44	CD133	VDR	Grade	FIGO Stage
CD44	1	0.2978 ***	0.6496 ***	0.3930 ***	0.4028 ***
CD133	0.2978 ***	1	0.4127 ***	−0.08114	−0.07031
VDR	0.6496 ***	0.4127 ***	1	0.4170 ***	0.4331 ***
Grade		−0.08114	0.4170 ***	1	0.3875 ***
FIGO stage	0.4028 ***	−0.07031	0.4331 ***	0.3875 ***	1

***, *p* (two-tailed) < 0.001 (n = 131).

## Data Availability

Data is contained within the article.
